# Testing relationship recognition in wild jackdaws (Corvus monedula)

**DOI:** 10.1038/s41598-019-43247-x

**Published:** 2019-04-30

**Authors:** Victoria E. Lee, Guillam E. McIvor, Alex Thornton

**Affiliations:** College of Life and Environmental Sciences, University of Exeter, Penryn Campus, Penryn, Cornwall TR10 9FE UK

**Keywords:** Animal behaviour, Behavioural ecology

## Abstract

According to the social intelligence hypothesis, understanding the challenges faced by social animals is key to understanding the evolution of cognition. In structured social groups, recognising the relationships of others is often important for predicting the outcomes of interactions. Third-party relationship recognition has been widely investigated in primates, but studies of other species are limited. Furthermore, few studies test for third-party relationship recognition in the wild, where cognitive abilities are deployed in response to natural socio-ecological pressures. Here, we used playback experiments to investigate whether wild jackdaws (*Corvus monedula)* track changes in their own relationships and the relationships of others. Females were presented with ‘infidelity simulations’: playbacks of their male partner copulating with a neighbouring female, and their male neighbour copulating with another female, against a congruent control. Our results showed substantial inter-individual variation in responses, but females did not respond more strongly to infidelity playbacks, indicating that jackdaws may not attend and/or respond to relationship information in this experimental context. Our results highlight the need for further study of relationship recognition and other cognitive traits that facilitate group-living in the wild, particularly in non-primates and in a wider range of social systems.

## Introduction

The social intelligence hypothesis posits that the sophisticated cognitive abilities seen in some species may have arisen due to the selection pressures associated with group living^1,2^. Several studies provide support for the social intelligence hypothesis, linking cognitive performance or brain size measures with various aspects of sociality^2–7^. However, other studies have shown conflicting results^8–10^ (see^11,12^ for a detailed discussion) and the social intelligence hypothesis remains controversial. To determine whether social life favours the evolution of associated cognitive abilities, it is necessary to understand how these cognitive abilities help individuals to navigate a dynamically changing social world.

Social species must solve ecological challenges within a social context^12,13^. In these cases, the ability to recognise other group members and remember past interactions allows individuals to predict (and potentially manipulate) others’ behaviour^14^. Although obtaining, processing and applying this knowledge is likely to be cognitively demanding^5,13,15^, individuals who are more socially competent may derive fitness benefits as a result^16–18^. In social groups where relationships persist over time, being able to track the relationships of other group members can be useful in predicting the outcomes of interactions^14^. Knowledge of third-party relationships might allow individuals to adjust their own behaviour appropriately to avoid conflict^19–22^, solicit and provide support during agonistic interactions^5,23–27^, and take advantage of mating opportunities^28^. Third-party relationship recognition has been demonstrated in several primate species, originally leading some authors to suggest that this ability may be confined to the primate order^29,30^. Observations of agonistic interactions indicate that bonnet macaques (*Macaca radiata*) solicit support from individuals who are higher-ranking than their opponent^23^ and chimpanzees (*Pan troglodytes*) will modify their recruitment screams depending on the dominance rank of bystanders^26^. Playback experiments also provide evidence that primates track third-party relationships. For instance, vervet monkeys *Chlorocebus aethiops pygerythrus*^20^ and chacma baboons *Papio hamadryas ursinus*^19^ respond to simulated reversals in the existing dominance hierarchy, demonstrating an understanding of the dominance relationships between other group members. Chimpanzees (*P*. *troglodytes*) will avoid aggressive individuals who are socially bonded to their former opponent, for several hours following an agonistic encounter^21^; and male baboons (*P*. *hamadryas ursinus*) track consortships between other males and females in order to obtain sneaky matings^28^. In vervet monkeys (*C*. *aethiops*), playbacks of infant distress calls cause nearby females to look towards the infant’s mother, demonstrating recognition of mother-offspring relationships within the social group^31^.

Few studies have investigated third-party relationship recognition in non-primates, despite many other species living in complex societies where this ability is expected to be useful. For example, hyenas (*Crocuta crocuta*) live in complex social groups with multiple hierarchically structured matrilines, similar to many primate societies^14^. Hyenas will join conflicts to support the higher-ranking individual even if the subordinate member of the fighting dyad is more aggressive, implying knowledge of the dominance relationships that exist in the group^24^ (but see^32^). Not only is it important to examine a diverse range of species, but also a diversity of social systems – for instance, little is known about the value of third-party relationship recognition in monogamous systems. Among birds, monogamy is the most common social system and has been argued to be central to the evolution of avian cognition^5^, although little is known about the cognitive demands associated with maintaining long-term pair bonds. Furthermore, many monogamous bird species live in groups and form stable, individualised relationships with others in addition to their breeding partner^5^. Corvids exhibit this type of social system, and their sophisticated cognitive abilities make them ideal subjects for investigating the evolution of social cognition^33–35^. Many corvids form long-term pair bonds and live in colonies characterised by strict dominance hierarchies between bonded pairs^36^. Empirical evidence supports the idea that recognising social relationships is beneficial in corvid colonies^35,37^. For example, playback experiments show that captive ravens (*Corvus corax*) respond to dominance rank reversals, both within their own social group and in a neighbouring group^22^. Furthermore, observations of wild ravens indicate that victims will reduce the frequency of their distress calls during agonistic encounters, if the bonding partner of their aggressor is present in the vicinity; victims also call more frequently when their own kin are nearby^27^. Anecdotal reports suggest that rooks (*Corvus frugilegus*) engage in redirected aggression, where individuals are more likely to attack their aggressor’s partner, or the aggressor of their partner, after a fight^5^. Finally, ravens will intervene in the affiliative interactions of others that appear to be establishing a strong bond, which is likely to require knowledge of the relationships of group members^38^.

In the only experimental test of third-party relationship recognition in corvids to date, Massen *et al*.^22^ found that ravens (*C*. *corax*) become stressed and engage in more self-directed behaviour after hearing simulated encounters that violate their expectation of the existing dominance hierarchy within their own colony. Male subjects also exhibited decreased calling and attention behaviour following simulated rank reversals in a neighbouring group, suggesting that ravens deduce third-party relationships by observation alone. However, this study was conducted under controlled conditions using captive individuals, where subjects could observe interactions between conspecifics very frequently. Consequently, it is not clear to what extent these results reflect the cognitive abilities animals employ in the wild, where a greater number of stimuli compete for individual attention^15,39,40^. Furthermore, most of the research carried out under natural conditions has involved observations of naturally-occurring behaviour, and there is a lack of experimental evidence for third-party relationship recognition in the wild outside the primate order. To this end, a recent study by Pardo *et al*.^41^ describes the first experimental field test for third-party relationship recognition in a non-primate. This study found that acorn woodpeckers initiate defensive behaviour more quickly in response to calls from two birds from different social groups, compared to calls of two birds from the same social group, suggesting that individuals recognise group membership outside of their own social group. However, it is not clear to what extent this indicates knowledge of the dyadic relationship between the two callers, or whether it is possible that subjects were responding to the unfamiliar stimulus of two calls occurring together when those calls had only been heard separately in the past. Consequently, much remains to be determined as to the extent of third-party relationship recognition in non-primates in the wild.

To address this research gap, we conducted an experiment to test whether wild jackdaws (a social corvid, *Corvus monedula*) track changes in their own relationships and the relationships of other members of their social group. This ability is likely to be useful in jackdaw society: pairs form monogamous bonds and females assume the rank of their male partner in the breeding colony’s strict linear dominance hierarchy^42^. These hierarchies remain relatively stable over time due to high adult survivorship (c. 80%, although estimates vary) and low rates of ‘divorce’^43^. For jackdaws, tracking relationships within the colony may allow individuals to avoid conflict with more dominant pairs, especially considering that competition over nest sites can be intense^43,44^. Relationship tracking may also allow individuals to notice if their partner is engaging in extra-pair copulations. Jackdaws are typically considered to be sexually as well as socially monogamous^45,46^, with studies to date finding that extra-pair paternity is rare: it has been suggested that the high level of parental investment required to successfully raise offspring may prevent birds from seeking extra-pair copulations^46^. However, recent findings suggest that extra-pair copulations may not be as uncommon as previously thought^47^; it may therefore pay females to track their partner’s behaviour.

**Figure 1:**
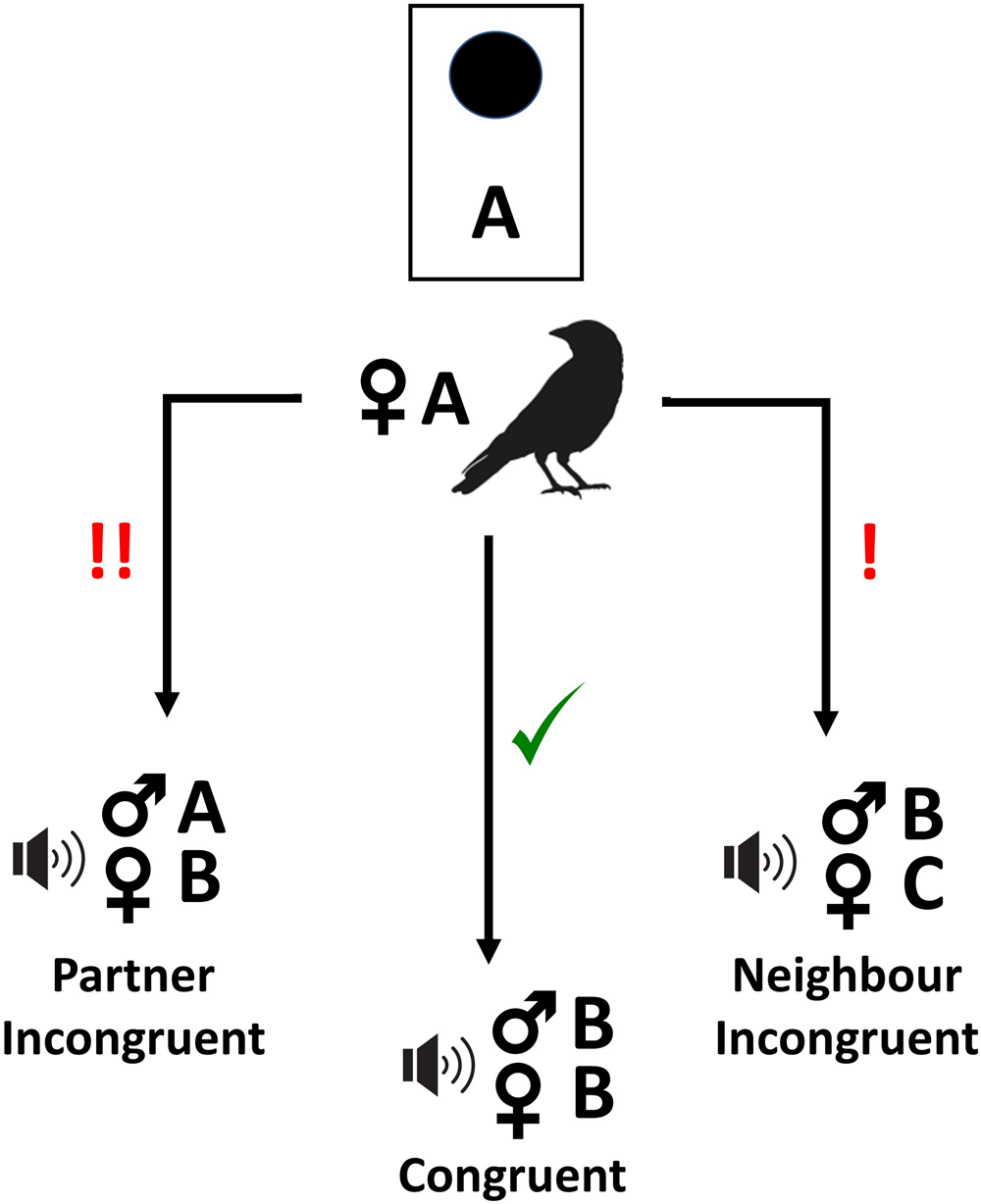
Experimental setup for each nest box. The focal female from nest box A heard three playback presentations. In the ‘Congruent’ control treatment, focal female A heard a playback simulating a copulation event between the neighbouring male from nest box B and the female from nest box B (his usual partner). The focal female (A) was expected to show a weak response to this playback, denoted by a green tick mark. In the ‘Neighbour Incongruent’ treatment, focal female A heard a playback simulating a copulation event between the neighbouring male from nest box B and the female from nest box C (not his usual partner). The focal female (A) was expected to show a stronger response to this playback as it violated expectations, denoted by a red exclamation mark. In the ‘Partner Incongruent’ treatment, the focal female (A) heard a playback simulating a copulation event between her own partner (male from nest box A) with the neighbouring female from nest box B. This playback was expected to elicit the strongest response from focal female A, denoted by two red exclamation marks.

Following the ‘violation of expectation’ paradigm employed in similar studies^20,22,28^, we used playback experiments to investigate whether female jackdaws respond to simulations of male infidelity. During mating, including extra-pair copulations, male jackdaws give loud copulation calls^48^. In a recent study combining acoustic tracking and video surveillance, male jackdaws were recorded emitting copulation calls at the same time as the female was alone on the nest^47^, suggesting that males do engage in extra-pair copulations and that this should be an ecologically relevant stimulus for the female. Furthermore, in our study population, intruder males are occasionally seen entering nest boxes and attempting to copulate with the incubating female (*pers*. *obs*.). Although it is not yet known whether male copulation calls encode information about caller identity, all other jackdaw vocalisations studied to date have been shown to be individually distinct (food calls^49^, contact calls^50^ and alarm calls^51^). Using playbacks of male contact calls and copulation calls in conjunction with female contact calls, we simulated mating events occurring during the egg-laying period of the breeding season, when copulation calls are heard most frequently in the colony (*pers*. *obs*.). Contact calls were included to ensure that playback sequences simulated interactions between individuals: contact calls are individually distinctive^50^ and typically accompany jackdaw copulation events. We used three playback treatments to test whether females track changes in their own relationships and the relationships of other colony members. In the ‘Partner Incongruent’ treatment, the playback simulated the focal female’s partner copulating with a female from a neighbouring nest, and this was expected to elicit a strong response from the focal female. A ‘Neighbour Incongruent’ treatment was designed to test third-party relationship recognition and simulated the male from a neighbouring nest copulating with another female who was not their usual partner. This was predicted to elicit an intermediate response from the focal female, as it violates expectations but does not involve the focal female’s own partner. Using a within-subjects design (Fig. 1), the responses of focal females to both ‘Incongruent’ playbacks were compared to a ‘Congruent’ control predicted to elicit a neutral response (playback of a neighbouring male copulating with their usual partner).

## Methods

### Ethics Statement

This experiment was carried out with approval from the University of Exeter research ethics committee (2015/974) and following the ASAB Guidelines for the Treatment of Animals in Behavioural Research and Teaching^52^. Although no birds were handled as part of this study, subjects had been previously captured and ringed by qualified bird ringers licensed by the British Trust for Ornithology and UK Home Office (project licence 30/3261).

### Study Population

This experiment was conducted during the 2015–2017 breeding seasons using free-living nest box populations of jackdaws, at three study sites in Cornwall, UK: a village churchyard (Stithians 50°11′26″N, 5°10′51″W; 33 nest boxes), an active farmyard (Pencoose Farm 50°11′56″N, 5°10′9″W; 35 nest boxes), and at the University of Exeter’s Penryn campus (50°17′32″N; 5°11′96″W; 11 nest boxes).

### Playback Experiments

#### Audio Recordings

Nest boxes occupied by breeding jackdaws were fitted with hidden CCTV cameras early in the nest-building phase (late March-early April). A subset of nest boxes selected for this experiment were also fitted with lapel microphones (n = 30). Focal nest boxes were selected with at least one marked individual, and with at least two nearby neighbouring pairs (within 50 m). This was to ensure that neighbours’ contact and copulation calls used in playbacks would be familiar and ecologically relevant stimuli for the focal female.

Audio recordings were made early in the morning (start time: 0700–0900) during late March and early April, when birds were engaged in nest building and copulation. Video recordings were made with digital video recorders (JXD 990) and audio recordings made with multitrack PCM recorders (Olympus LS-100 & Tascam DR-100MKII). Recordings were made daily as required to obtain the necessary vocalisations for use in playback experiments. Each recording ran for 3.5 hours. For some subjects, copulation and contact calls were extracted from recordings obtained during previous seasons (2013–2015) using an identical protocol.

#### Call extraction

Clear exemplars of contact calls and copulation calls with minimal background noise were extracted from nest box audio recordings and normalised for amplitude using Audacity (www.audacityteam.org). The context of vocalisations and the identity of the caller were ascertained using nest box videos collected alongside the audio recordings. In cases where females vocalised during copulation, female calls were removed from the audio track, leaving only the male copulation call. Extracted calls were arranged into playback files containing a male contact call, followed by a female contact call, followed by a male copulation call, to simulate a copulation event (see Fig. S1 in Supplementary Material). Calls occurred at 2 s intervals to simulate natural calling, and male copulation calls varied in length – this variation was retained to avoid excessively editing the acoustic stimulus and potentially altering important aspects of call structure, but playback duration was later controlled for statistically (see *Statistical Analysis)*. Because of the limited number of suitable copulation call recordings, and the variation in copulation call duration within and between males, some copulation calls appeared in multiple playback trials. Focal females heard the same copulation call from the male neighbour in the Congruent and Neighbour Incongruent treatments, to ensure consistency across the experiment and minimise the potential confounding effects of call duration. Contact calls were not repeated across playback trials.

#### Experimental Design

This experiment followed a repeat measures design with each focal female (Female A) being assigned three playback files (one for each of the experimental treatments) as follows:

- Congruent treatment: Neighbour Male B ‘copulating’ with Neighbour Female B.
- Partner incongruent treatment: Partner Male A ‘copulating’ with Neighbour Female B.
- Neighbour incongruent treatment: Neighbour Male B ‘copulating’ with Neighbour Female C (Fig. 1).

All experimental trials occurred soon after eggs were laid by the focal female, when females were motivated to remain in the nest box but copulation calls were still being heard frequently around the nesting colony. The order in which focal females received each playback treatment was counterbalanced as far as possible, to ensure a matched design across the experiment. At least 24 hours elapsed between trials for a given focal nest box. All trials were carried out between 09:00 and 18:30, to coincide with peak activity times of the birds^44^.

We carried out 28 trials across three sites in 2015–2017, at 10 focal nest boxes (two trials were discarded due to camera failure). This was the maximum sample size that could be achieved in this case, due to the limited number of nest boxes with at least two close neighbours and the difficulties in obtaining enough calls from these pairs. All females were colour-ringed, except one bird whose partner was colour-ringed enabling identification of individuals at the nest box. Trials were not carried out in the same area of the colony in the same year. In cases where trials were carried out in the same area in subsequent years, neighbouring birds from previous years were not included in the experiment as focal individuals.

#### Experimental trials

Prior to trials, a remote-controlled FoxPro Fury 2 loudspeaker (disguised with vegetation to avoid any neophobic responses) was attached to a tripod and placed approximately two-thirds of the distance between the focal nest and the neighbour nest (mean distance 13.6 m between focal nest box and loudspeaker, range 8–21 m). The loudspeaker was set up in the same location for all trials at a nest box. Video recording equipment was also set up (DVR JXD 990) to record female behaviour inside the focal nest box and neighbouring nest box.

Following setup, the experimenter returned to a concealed location a minimum of 50 m away. Playbacks only occurred after the focal female had remained undisturbed in the nest box for at least 5 minutes (no disturbance outside the nest box, female had not left the box or appeared at nest box entrance), and at least 5 minutes following the most recent visit by the male. A baseline period of at least 20 minutes elapsed between the female’s first return to the nest box and presentation of the playback stimulus, to allow focal pairs to return to normal behaviour after setting up equipment.

### Behavioural Analysis

Footage of focal females was analysed using BORIS^53^. The frequency and duration of behaviours exhibited by the focal female were recorded for the 2-minute period following the start of each playback presentation. These included: (i) categorical primary response to playback (looking at the nest box entrance, peeking out of the nest box, or leaving the nest box); (ii) time spent looking at the nest box entrance and peeking out of the nest box. All playbacks were conducted at least 5 minutes after the last visit by the male. There were 5 instances where males returned to the nest box in the two minutes following the playback, and in these cases all female behaviours occurring during and after the male’s visit were discounted.

Twenty percent of videos were analysed by a second coder who was blind to treatment. Inter-rater reliability was analysed using a two-way intraclass correlation coefficient (ICC) and indicated a high level of agreement between coders for all behaviours analysed (time spent looking at entrance in the post-playback period: ICC = 0.98, p < 0.001; time spent peeking in the post-playback period: ICC = 0.87, p = 0.006. In all cases, both coders agreed on the categorical primary response to the playback).

### Statistical Analysis

All analyses were carried out in R v3.4.3^54^ with models were built using lme4^55^ and ordinal^56^. Model plots were examined to ensure that assumptions were met (homogeneity and normality of residuals), and minimum adequate models were obtained via log-likelihood ratio tests.

#### Behavioural response to playback

In all cases females looked towards the entrance in response to the playback, but some individuals subsequently went on to peek out of the nest box entrance or leave the nest box. The extent of female response was analysed using a cumulative link mixed model (CLMM) using female behaviour (LOOK/PEEK/EXIT) as an ordinal response term. In the model, leaving the nest box was considered the strongest response to the playback (EXIT = 3), followed by peeking out of the nest box from a standing position (PEEK = 2), with looking at the nest box entrance from a seated position taken to be the weakest response (LOOK = 1). Treatment (congruent, partner incongruent or neighbour incongruent) and trial number (1–3) were included as fixed effects and female ID as a random term. The effect of female identity on response was analysed using log-likelihood comparison between the minimal model and a cumulative link model without the random factor^57^. Four trials were excluded from the analysis as the male returned to the nest box prior to the end of the playback, likely influencing female response.

#### Time spent looking and peeking following playback

For the two-minute period following the start of the playback, the time that each female spent looking at the nest box entrance and/or peeking out of the nest box was analysed using a general linear mixed model (GLMM) with a Gaussian error distribution. Treatment (congruent, partner incongruent or neighbour incongruent), trial number (1–3) and length of playback were included as fixed effects with focal female ID as a random term. Of the 28 trials, 6 were discarded as the male returned to the nest within two minutes of the playback. One focal female responded to the playback by leaving the box immediately in all three trials, and these were likewise excluded from the analysis. An influential data point was also removed from the model following examination of Cook’s distances: in this case, the focal female spent the full two-minute period looking at the nest box entrance, but was also facing the nest box entrance when the playback started (and therefore may not have represented a reliable response to the playback).

## Results

**Figure 2:**
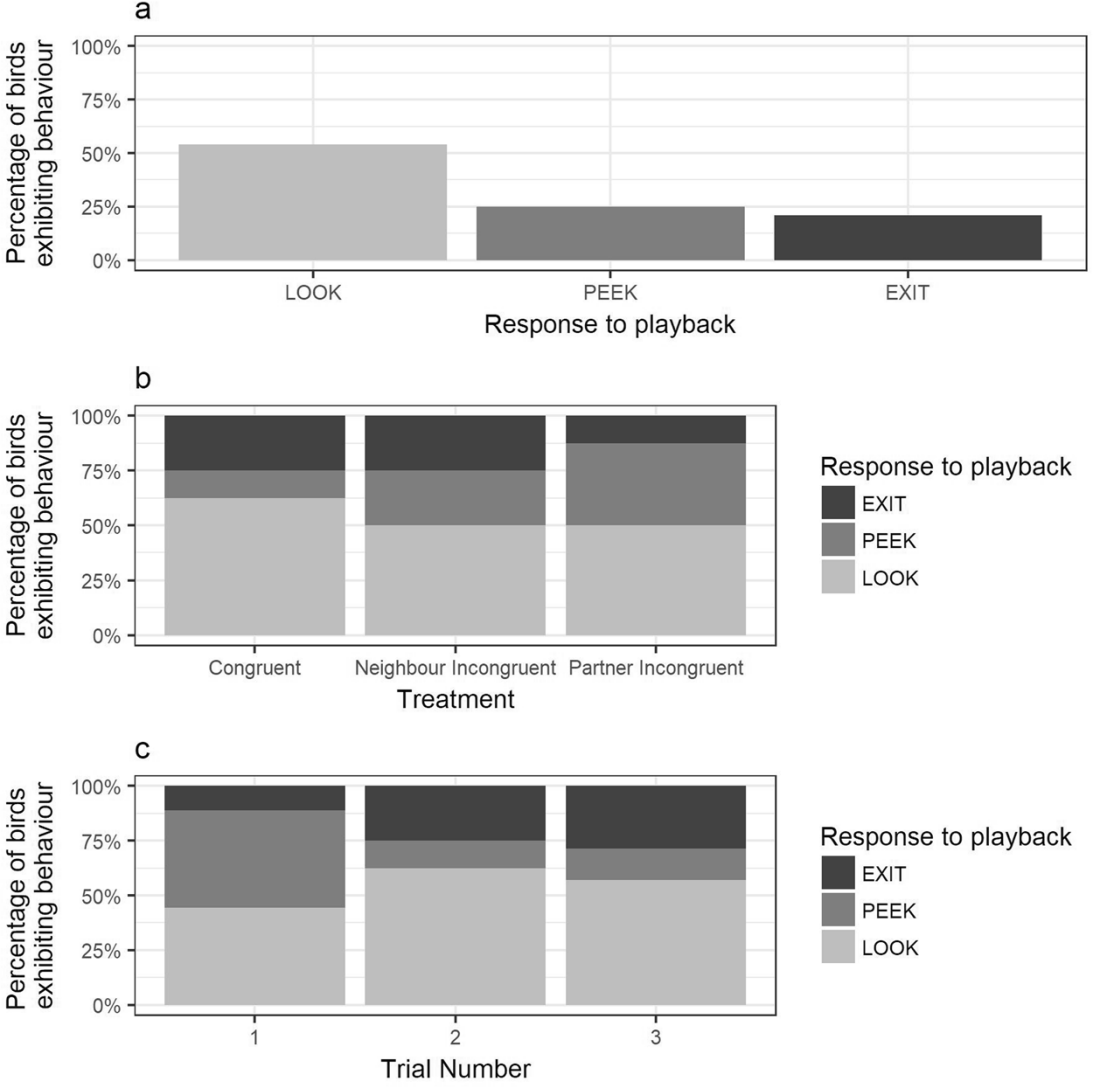
Barplots showing behavioural responses of females to playback treatments: looking at the nest box entrance from an incubating position (LOOK, light grey bars); peeking out of the nest box entrance from a standing position (PEEK, mid-grey bars), and leaving the nest box (EXIT, dark grey bars). (**a**) Percentage of females exhibiting each response across all trials; (**b**) percentage of females exhibiting each behaviour by treatment (congruent, neighbour incongruent, partner incongruent); (**c**) percentage of females exhibiting each behaviour by trial number (1–3, treatment presentations counterbalanced across trials).

In all cases, females showed some form of response to the playback. These responses ranged from looking at the nest box entrance from a seated position during incubation (“LOOK”, 54% of cases), moving to look out of the nest box entrance (“PEEK”, 25% of cases) and leaving the nest box (“EXIT”, 21% of cases) (see Fig. 2). On no occasion did females vocalise in response to the playback. During the post-playback observation period, there were two occasions when an intruding male (not the focal female’s partner) entered the nest box and attempted to copulate with the focal female. These incidents both occurred during the first trial at the nest boxes in question and approximately half an hour after the playback presentation; once following a ‘Partner Incongruent’ playback (2015) and once following a ‘Congruent’ playback (2017). Intrusions by other males were not observed during any other trials, either before or after the playback presentation.

### Behavioural response to playback

**Figure 3:**
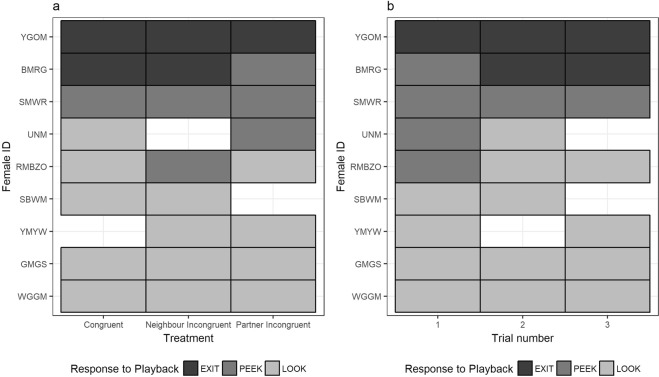
Tile plots showing responses of focal females to the three playbacks, according to (**a**) treatment (Congruent, Neighbour Incongruent, Partner Incongruent) and (**b**) trial number (1–3). Female ID (y-axis) shows colour-ring combinations of focal females. Tile colour corresponds to the behavioural response of the female to the playback: looking at the nest box entrance from an incubating position (LOOK, light grey bars); peeking out of the nest box entrance from a standing position (PEEK, mid-grey bars), and leaving the nest box (EXIT, dark grey bars). Blank tiles represent trials where a reliable measure of females’ initial response to the playback could not be obtained.

Females responded to playbacks by looking at the nest box entrance (LOOK), peeking out of the entrance from a standing position (PEEK) or leaving the nest box (EXIT). However, the likelihood of females exhibiting these behaviours was similar across treatment groups (CLMM: X^2^ = 1.21, df = 2, p = 0.55) and was not influenced by trial order (CLMM: X^2^ = 0.40, df = 2, p = 0.82) (Fig. 2, Table 1). Instead, response to playbacks was strongly influenced by the identity of the female (CLM: X^2^ = 12.3, df = 1, p < 0.001). For example, females that left the box in one trial were more likely to do so in subsequent trials (Fig. 3, Table 1).

**Table 1:** Output of CLMM investigating the effect of treatment (congruent, neighbour incongruent, partner incongruent) and trial number (1–3) on the ordinal response of females to the playback (LOOK = looking at nest box entrance, PEEK = peeking out of nest box entrance, EXIT = leaving the nest box).

Model parameters	β	SE	z-value	p-value
**Threshold (response)**				
LOOK|PEEK	1.47	2.74	0.54	
PEEK|EXIT	6.31	2.74	2.30	
**Treatment**				
**Congruent (reference)**				
Neighbour incongruent	1.56	1.80	0.86	0.39
Partner incongruent	−0.13	1.63	−0.08	0.94
**Trial number**				
**Trial 1 (reference)**				
Trial 2	−0.53	1.62	−0.33	0.74
Trial 3	−0.22	1.66	−0.13	0.89
**Random effects**			**Variance**	**SE**
*Female ID*			*31*.*89*	*5*.*65*

Congruent treatment and Trial 1 are the reference levels, n = 24 observations of 9 females. Values shown from full model, statistically significant effects are given in italics.

### Time spent looking/peeking following playback

**Figure 4:**
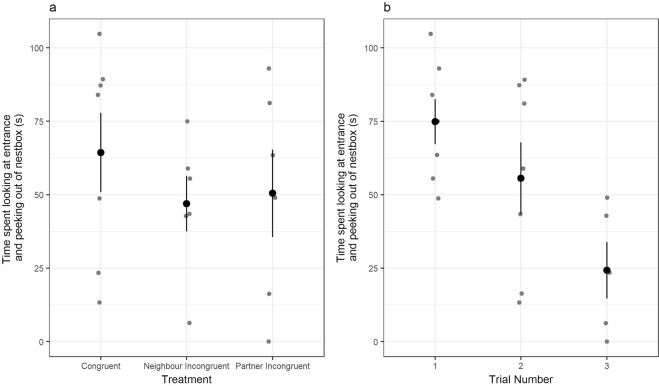
Plots showing the time spent by focal females looking at the nest box entrance or peeking out of the nest box entrance in the two minutes post-playback, by (**a**) treatment (congruent, neighbour incongruent, partner incongruent) and (**b**) trial number (1–3). Grey points represent individual data points (n = 19 observations of 8 females), and black points with error bars denote mean and standard error.

In the two-minutes following the start of the playback, females spent an average of 54 s (±7.3 s) either looking at or peeking out of the nest box entrance. The length of time that females spent looking at or out of the nest box entrance did not differ between treatments (GLMM: X^2^ = 0.58, df = 2, p = 0.75), and was not influenced by the duration of the playback (GLMM: X^2^ = 1.12, df = 1, p = 0.29). However, females spent less time looking and peeking following playbacks as trials progressed (GLMM: X^2^ = 10.13, df = 2, p = 0.006) (Fig. 4, Table 2).

**Table 2:** Output of GLMM investigating time spent looking at nest box entrance and peeking out of nest box entrance in the two minutes following the start of playback. Full model includes treatment, trial order and playback duration as fixed effects (statistically significant effects given in italics).

Fixed effect		β	SE	t-value
**Full model**				
Intercept		101.46	22.97	4.42
Treatment	Congruent (reference)			
	Neighbour incongruent	−7.27	13.0	−0.56
	Partner incongruent	−9.59	13.12	−0.73
*Trial order*	*Trial 1 (reference)*			
	*Trial 2*	*−16*.*72*	*12*.*31*	*−1*.*36*
	*Trial 3*	*−47*.*20*	*13*.*55*	*−3*.*48*
Playback duration		−1.33	1.24	−1.08

Congruent treatment and Trial 1 are the reference levels, n = 19 observations of 8 females.

## Discussion

We found no effect of treatment on jackdaws’ responses to playbacks, with females behaving in a similar manner following simulations of their partner’s infidelity, their neighbour’s infidelity and a congruent control. There were no significant differences in females’ initial response (looking at the nest box entrance, peeking out of the nest box entrance or leaving the nest box) or the duration of the response (time spent looking at, or out of, the nest box entrance). However, females appear to habituate to playbacks over time, as the length of time females spent investigating the stimulus (looking at or out of the nest box entrance) decreased over successive trials.

Each subject’s initial response to the playback (looking at the nest box entrance, peeking out of the nest box entrance or leaving the nest box) was strongly influenced by the identity of the individual. Inter-individual variation between females was significant, with females’ response during their first trial strongly predicting their response during subsequent trials, regardless of treatment. In terms of the duration of this response (time spent looking at/out of the nest box entrance), none of the test subjects behaved as predicted: we found no evidence of a stronger response to the ‘Partner Incongruent’ or ‘Neighbour Incongruent’ treatments compared to the ‘Congruent’ control. Two females looked/peeked for longer following the infidelity simulation of their partner compared to the control playback, but this may be because these subjects heard their partner’s infidelity simulation first. Overall, these results suggest that individual variation likely plays an important role in influencing subjects’ responses in these types of experiments, yet these individual differences are rarely examined or discussed explicitly in studies of cognition^58–60^.

Although these results do not provide any evidence that jackdaws track their own relationships and the relationships of others in their social group, this does not necessarily imply that jackdaws are incapable of third-party relationship recognition. Instead, it may be that birds simply failed to demonstrate this ability within the context of our experimental setup. The fact that females failed to respond to simulations of their own partner’s infidelity, as well as the infidelity of a male neighbour, is consistent with this possibility. There are several potential explanations as to why female responses did not differ between experimental treatments. Firstly, the experiment was carried out during an ecologically relevant period when birds were copulating at a high rate compared to other stages in the breeding attempt. It may be that if copulation calls are heard frequently around the colonies at this time, individuals attend to (or ignore) all copulation calls equally. Moreover, it is possible that females do not discriminate between the copulation calls of individual males (although jackdaw contact calls are individually distinct^50^, and were included in playback sequences to simulate interactions between individuals). Furthermore, if extra-pair copulations are extremely rare^45,46^, females may not perceive the playback stimulus as an ‘infidelity’. However, recent evidence^47^ and observations of intruder males in our own study population suggest that extra-pair copulations in jackdaws may occur more commonly than previously thought. For this reason, it seems that it would be beneficial for females to notice when their partner is copulating with another female. If females do perceive the playback stimulus as an ‘infidelity’, perhaps there is no advantage to females in acting on this information (e.g. by leaving the nest to gather more information, or to retaliate against their unfaithful partner^61^). In a similar experiment, Crockford *et al*.^28^ found that subordinate male baboons respond to playbacks of female copulation calls that were indicative of a recent consortship having ended, as these cues provide highly relevant information which may allow them to gain ‘sneaky’ matings. In our study it is possible that, if there is no direct fitness benefit to females, the social information indicating male infidelity is not attended to or acted upon to the same extent. The fact that nest intrusions occurred following two of the playback presentations (where another male entered the focal nest box and attempted to copulate with the resident female) raises the possibility that male jackdaws may eavesdrop on copulation events in a similar way to baboons^28^. Finally, if male infidelity does not reduce subsequent paternal care, there may be little cost to their female partner. Given the high degree of social monogamy in this species^46^, it may be that male extra-pair copulation does not merit a response from females. It would be interesting to determine whether male extra-pair copulation behaviour, or playback simulations of male infidelity, influence female behaviour over the long term (e.g. in terms of mate choice, see^62^).

Females showed habituation to playbacks over time, suggesting that there may be aspects of our experimental setup that were incongruent with naturally-occurring copulation events. For example, the timings of calls in the playback sequence may not be a reliable indication of two birds being in close proximity at the same time. Each playback sequence consisted of a male contact call and female contact call, followed by a copulation call from the same male (Fig. S1 in supplementary material). A pause of two seconds occurred between each call, which represents natural calling rates for individual birds (unpublished data). Playbacks were conducted when the area was quiet and no other birds were heard calling, but in busy areas of the colony where calling is generally frequent, it may be that the calls of multiple birds are frequently heard together without any direct interaction between callers. The fact that all playback calls were emitted from the same direction may have provided an additional cue that calls represent a social interaction; on the other hand, call direction may be difficult for a female jackdaw to discern from inside a nest box. Observations of female responses to naturally-occurring copulation events and male infidelity may shed light on why females failed to respond to our playbacks, and would be an important avenue for future study.

It could be that jackdaws are more likely to respond to relationship changes that influence agonistic encounters, such as changes in dominance rank. Jackdaw colonies are structured according to a linear dominance hierarchy, where females assume the rank of their male partner^42^. Pairs then compete for food and nest sites, with conflict over nesting cavities being particularly intense^43,44^. Recognising changes in dominance rank may be of fitness relevance to birds in allowing them to gain access to resources whilst avoiding conflicts that are potentially costly. Playback experiments have demonstrated that primates recognise changes in dominance rank^19,20^, and hyenas also appear to apply knowledge of third-party relationships during agonistic interactions^24^ (but fail to demonstrate this ability in other contexts^32^). Unfortunately, jackdaws do not give dominance calls, which would make an experimental test of knowledge of third-party ranks logistically challenging. Other corvids have been shown to respond to simulated changes in dominance rank, both within their own social group and a neighbouring group^22^. However, this study was conducted in captivity with small groups of birds housed in close proximity. Birds therefore had extensive opportunities to learn about social relationships by observing frequent interactions between all group members; it is currently unknown whether these opportunities occur similarly under natural conditions. Therefore, the extent of third-party relationships knowledge in the wild, and the contexts in which corvids apply this knowledge, remains to be determined.

This study presents one of the first experimental tests of third-party relationship recognition in a non-primate under natural conditions. To date, only one other field experiment has been conducted on birds, and suggests that acorn woodpeckers are aware of which individuals make up neighbouring groups^41^. However, it is unclear whether the act of calling together in woodpeckers provides any information about the nature of the dyadic relationship between callers. Here, we used copulation calls, which are directed at specific individuals during a specific type of social interaction, to investigate dyadic and third-party relationship representation. We found no evidence that jackdaws track their own relationships and the relationships of other individuals in their social group. However, we cannot rule out that jackdaws possess this ability, as none of the test subjects responded in a manner consistent with the experimental predictions. Moreover, due to the difficulties in obtaining a sufficient number of calls from close neighbours in the experimental colonies, our sample size (n = 10) is modest (see Methods). Our sample size is in line with similar studies of corvids in captivity, both for tests of social cognition and cognitive abilities more generally^22,63–67^. It could be that under natural conditions, where subjects’ attention is divided and there are more confounding environmental variables, larger sample sizes are required to detect an effect. This emphasises the need to complement research in the laboratory with rigorous field studies addressing questions related to social cognition.

A growing body of research, both observational and experimental, shows that species that live in complex societies possess knowledge of third-party relationships and other socio-cognitive abilities considered to be relatively ‘sophisticated’. To date, many of these studies have been carried out using captive populations, with field studies mostly confined to primates. More studies are needed in a wider range of species and social systems, especially in a field context where findings may be more likely to accurately reflect the cognitive processes animals use to solve real-world socio-ecological challenges^39,40^. Studies of this kind would make a valuable contribution to our understanding of social cognition in different species, and how these abilities help individuals to navigate a changing social world.

## Supplementary information

Supplementary Material

## Data Availability

Data and R scripts associated with this study are available in the Figshare repository (10.6084/m9.figshare.7825943).
