# Supplementary material for: Testing relationship recognition in wild jackdaws (Corvus monedula)
Source: Sci Rep. 2019 Apr 30;9:6710. doi: 10.1038/s41598-019-43247-x (PMC6491552; doi:10.1038/s41598-019-43247-x)
Supplement: Supplementary file 1 — Supplementary Material [file 41598_2019_43247_MOESM1_ESM.pdf]

Testing relationship recognition in wild jackdaws (Corvus monedula)

Victoria E. Lee[a*], Guillam E. McIvor[a] & Alex Thornton[a]

[a]College of Life and Environmental Sciences, University of Exeter, Penryn Campus, Penryn, Cornwall, UK TR10 9FE.

*Email correspondence: vel202@exeter.ac.uk

Supplementary Material

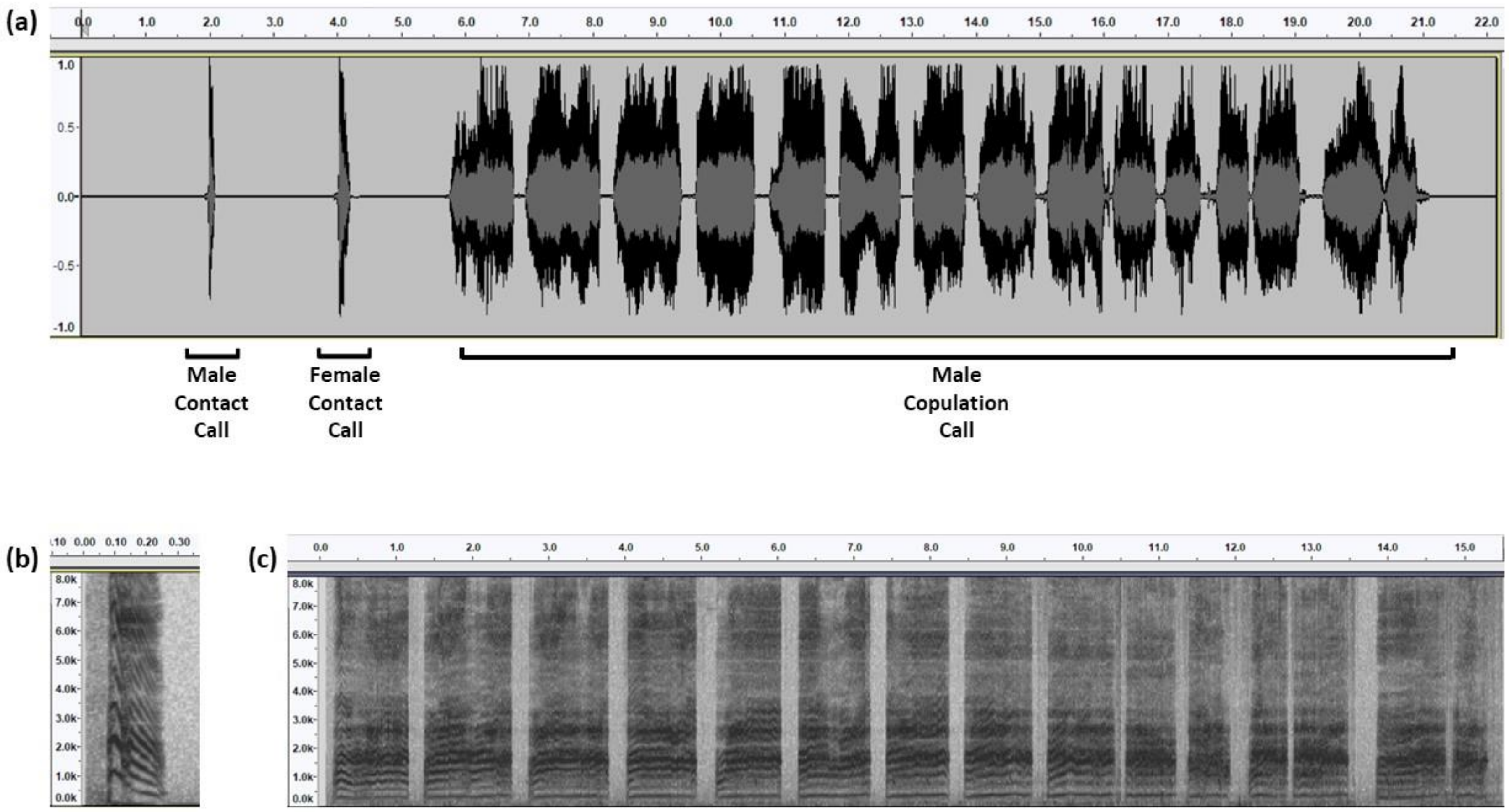

Fig. S1: Diagram showing structure of playbacks. a) Waveform of a playback track simulating infidelity at a neighbouring nest box. Each sequence was comprised of a male contact call, a female contact call and a copulation call from the same male. Male copulation calls varied in length; this variation was retained to avoid over-editing important call features and was later controlled for statistically; b) Spectrogram of a jackdaw contact call; c) Spectrogram of a jackdaw copulation call.
